# Detection of Negative Stress through Spectral Features of Electroencephalographic Recordings and a Convolutional Neural Network

**DOI:** 10.3390/s21093050

**Published:** 2021-04-27

**Authors:** Arturo Martínez-Rodrigo, Beatriz García-Martínez, Álvaro Huerta, Raúl Alcaraz

**Affiliations:** 1 Research Group in Electronic, Biomedical and Telecommunication Engineering, Facultad de Comunicación, University of Castilla-La Mancha, 16071 Cuenca, Spain; 2 Instituto de Tecnologías Audiovisuales de Castilla-La Mancha, University of Castilla-La Mancha, 16071 Cuenca, Spain; Alvaro.Huerta@uclm.es; 3 Departamento de Sistemas Informáticos, Escuela Técnica Superior de Ingenieros Industriales, University of Castilla-La Mancha, 02071 Albacete, Spain; Beatriz.GMartinez@uclm.es; 4 Instituto de Investigación en Informática de Albacete, University of Castilla-La Mancha, 02071 Albacete, Spain; 5 Research Group in Electronic, Biomedical and Telecommunication Engineering, Escuela Politécnica de Cuenca, University of Castilla-La Mancha, 16071 Cuenca, Spain; raul.alcaraz@uclm.es

**Keywords:** convolutional neural networks, electroencephalography, power spectral density, negative stress

## Abstract

In recent years, electroencephalographic (EEG) signals have been intensively used in the area of emotion recognition, partcularly in distress identification due to its negative impact on physical and mental health. Traditionally, brain activity has been studied from a frequency perspective by computing the power spectral density of the EEG recordings and extracting features from different frequency sub-bands. However, these features are often individually extracted from single EEG channels, such that each brain region is separately evaluated, even when it has been corroborated that mental processes are based on the coordination of different brain areas working simultaneously. To take advantage of the brain’s behaviour as a synchronized network, in the present work, 2-D and 3-D spectral images constructed from common 32 channel EEG signals are evaluated for the first time to discern between emotional states of calm and distress using a well-known deep-learning algorithm, such as AlexNet. The obtained results revealed a significant improvement in the classification performance regarding previous works, reaching an accuracy about 84%. Moreover, no significant differences between the results provided by the diverse approaches considered to reconstruct 2-D and 3-D spectral maps from the original location of the EEG channels over the scalp were noticed, thus suggesting that these kinds of images preserve original spatial brain information.

## 1. Introduction

Nowadays, one of the major issues in advanced societies is negative stress, also known as distress, given its detrimental influence on health of people who suffer from it [1,2]. Inhabitants in developed countries are surrounded by an economic and social pressure and a frenetic rhythm of life that leads them to a continuous state of anxiety and nervousness [3]. Furthermore, the current coronavirus pandemic has contributed to generally increasing the level of uncertainty and distress in the global population, with multifaceted drastic repercussions for people’s lives [4]. Short-term negative stress appears as a fight or flight reaction for self-protection and integrity of the organism, and it may not be a risk factor for health [5]. However, a long-term exposure to distressful conditions has demonstrated the production of serious negative effects on physical and mental health, causing, or even aggravating, several disorders related to cerebral, immune and endocrine systems [6,7]. Consequently, the accurate detection and regulation of distress could be essential to maintaining a healthy functioning [8].

Negative stress produces a series of measurable alterations on different physiological systems, such as the brain [9]. Brain activity can be quantified by means of electroencephalographic (EEG) recording, which represents the electrical activity originated under the scalp due to neural connections. The brain is the physiological system firstly responding against any stimulus, and then this response spreading to the rest of the peripheral organs by means of the central nervous system [10]. Therefore, the assessment of EEG signals may reveal more relevant information than secondary effects of the brain’s activity in the rest of the body [10]. This could be why the study of EEG recordings is receiving increased interest for the recognition of emotional states in recent years [11].

Traditionally, EEG recordings have been studied from a frequency perspective, analyzing brain activity in different spectral bands [12]. Indeed, it is widely known that emotional processes may induce changes in the cognitive state of an individual, which are accompanied by alterations in the brain’s oscillatory activity [13]. The information of interest in emotional processes occurs between 4 and 45 Hz, corresponding to theta (θ), alpha (α), beta (β) and gamma (γ) frequency sub-bands [14]. To analyze this information, power spectral density (PSD) is usually computed using different algorithms, such as Fast Fourier transform or Welch’s periodogram [12]. Then, power features are extracted from the different frequency sub-bands and combined using machine learning methodologies, such as k-nearest neighbor (k-NN) [15], support vector machine (SVM) [16,17], and Bayes neural networks [18]. However, most previous works only focused on how to combine these single-frequency parameters, without exploring spatial information collected from locations of the electrodes over the scalp [19].

Recently, in addition to machine learning methods, deep learning algorithms have also been used to diagnose some mental disorders, such as epilepsy [20,21], dementia [22], depression [23] or Parkinson [24]. In this respect, convolutional neural networks (CNN) are the most-used techniques within the wider context of deep-learning [25], as well as in the emotion recognition field [26]. Unlike traditional machine learning methods, CNN automatically learns complex features using different convolutional filters and combining the weights to predict class membership. According to the literature, most of the studies related to emotion recognition with CNN used 2-D EEG spectrograms as input data [27,28,29]. However, these spectrograms represent the information from a single channel, with each brain region being evaluated separately. From this perspective, the global coordinated brain information is ignored even when it has been corroborated that the brain works as a network, and mental processes are based on the synchronized performance of different areas [30,31]. Hence, for a thorough assessment of the underlying brain dynamics under different emotions, simultaneous analysis of all brain regions has been suggested [32,33].

To take advantage of that global brain information, the present work evaluates, for the first time, 2-D and 3-D spectral images constructed from simultaneous 32 channels typically acquired by EEG recordings. More precisely, a well-known CNN-based model, such as AlexNet, is proposed to discern these images from emotional states of calm and distress. Moreover, transfer learning (TL) has proven to be an effective methodology in many applications to palliate some drawbacks originated when a CNN is trained from the beginning [34]. Thus, several CNN models conserving the hyper-parameters from the original pre-trained AlexNet and with randomized initial hyper-parameters have been analyzed in the present work. Additionally, since there is not a gold-standard method regarding the rearrangement of the common location of the EEG electrodes over the scalp into a 2-D map [35], diverse approaches found in the literature have been compared.

The structure of the paper is as follows. Section 2 describes the analyzed database, as well as how the EEG signals were preprocessed and their power spectral density was computed. The diverse options considered for the rearrangement of EEG electrode locations into 2-D maps, the trained CNN models and the experimental protocol are also included in this section. The results obtained are presented in Section 3 and discussed in Section 4. Finally, conclusions extracted from this study are summarized in Section 5.

## 2. Materials and Methods

### 2.1. Dataset

The EEG signals analyzed in this study were extracted from the Database for Emotion Analysis using Physiological Signals (DEAP) [36]. This publicly available database consists of a total of 1280 samples of various emotional states, obtained from 32 participants (50% males, mean age 26.9 years). Each subject visualized 40 one-minute length videoclips with emotional content, while EEG signals and other physiological variables were recorded. After each visualization, participants rated their emotional state by means of self-assessment manikins (SAM), which are a graphical representation of nine levels of intensity of different emotional parameters. More precisely, the ratings of two parameters, called valence (i.e., the degree of pleasantness or unpleasantness of a stimulus) and arousal (i.e., the level of activation or deactivation provoked by a stimulus), were considered in this study. Although the whole scales of valence and arousal were covered during the creation of the DEAP dataset, only samples corresponding to calm and distress emotions were selected for the present study. In accordance with previous works [37,38], samples from the distress group were selected as those with a valence lower than 3 and an arousal higher than 5. On the other hand, the calm group was formed from samples with a valence between 4 and 6, and an arousal level lower than 4. Hence, a total of 122 trials of distress and 137 of calm were finally analyzed.

### 2.2. Preprocessing of the EEG Recordings

The EEG recordings were obtained from 32 channels distributed over the scalp according to the international standard 10–20 system for electrodes location [39]. Before further analysis, the raw EEG signals were preprocessed in order to eliminate nuisance and interferences blurring neural information. Precisely, the Matlab toolbox EEGLAB, specifically created for processing EEG recordings, was applied for this purpose [40]. The signals were firstly downsampled from 512 to 128 Hz, and a new reference based on the average potential of all electrodes was established. Later, high-pass and low-pass forward/backward filters were applied at 3 and 45 Hz, respectively, with the aim of maintaining the frequency sub-bands of interest in the EEG spectrum [14]. These filtering approaches also eliminated baseline and power line interferences. Then, artifacts and other interferences not eliminated in previous steps were rejected by means of a blind source separation technique, called independent component analysis (ICA). It is based on the computation of independent components, such that those identified as artifactual were discarded, and only the information related to brain activity was maintained [41]. Briefly, ICA was applied for the removal of artifacts derived from physiological (eye blinks, facial movements, or heart bumps) and technical sources (electrode-pops, or bad contacts of the electrodes on the scalp). Finally, highly contaminated channels were eliminated and reconstructed by interpolating the adjacent electrodes [42]. Although these noisy signals were identified before ICA procedure, the interpolation was done after that step to avoid the nonlinearities derived from the influence of interpolation on ICA decomposition [43]. Hence, the rejection of artifacts was not affected by the existence of the noisy channels replaced by interpolation [43].

### 2.3. Power Spectral Density Computation

Although the EEG signals collected in the DEAP database had a duration of 60 s, only the last 30 ones were used for power computation from frequency sub-bands, as in previous studies [36,37]. This segment for each EEG recording was then divided into six nonoverlapped epochs of 5 s of length, such that a total number of 822 (137 trials × 6 epochs/trial) and 732 (122 trials × 6 epochs/trial) excerpts were analyzed for emotional states of calm and distress, respectively. PSD was computed from every segment using a Welch’s periodogram, with a Hamming window of 2 s-length, 50% of overlapping between adjacent windows and a resolution of 256 points. Then, the power for every EEG channel was individually obtained for each frequency sub-band (θ, α, β and γ) and for the whole band (4–45 Hz) as the area under the PSD curve within the corresponding frequency band. In the case of the frequency sub-bands θ, α, β and γ, the resulting power was normalized by the one obtained for the whole band, with the purpose of preserving the variations between subjects [17]. Equations (1)–(5) describe the power computation for all the band, and for each frequency sub-band, respectively, with $P_{w}\left(f\right)$ the PSD estimated for each EEG excerpt.

(1) $$P_{t}=\sum_{f=4\;\mathrm{Hz}}^{f=45\;\mathrm{Hz}}\left|P_{w}\left(f\right)\right|$$

(2) $$P_{\theta }=\frac{1}{P_{t}}\sum_{f=4\;\mathrm{Hz}}^{f=8\;\mathrm{Hz}}\left|P_{w}\left(f\right)\right|$$

(3) $$P_{\alpha }=\frac{1}{P_{t}}\sum_{f=8\;\mathrm{Hz}}^{f=13\;\mathrm{Hz}}\left|P_{w}\left(f\right)\right|$$

(4) $$P_{\beta }=\frac{1}{P_{t}}\sum_{f=13\;\mathrm{Hz}}^{f=30\;\mathrm{Hz}}\left|P_{w}\left(f\right)\right|$$

(5) $$P_{\gamma }=\frac{1}{P_{t}}\sum_{f=30\;\mathrm{Hz}}^{f=45\;\mathrm{Hz}}\left|P_{w}\left(f\right)\right|$$

### 2.4. Rearrangement of EEG Channels in 2-D and 3-D Maps

**Figure 1:**
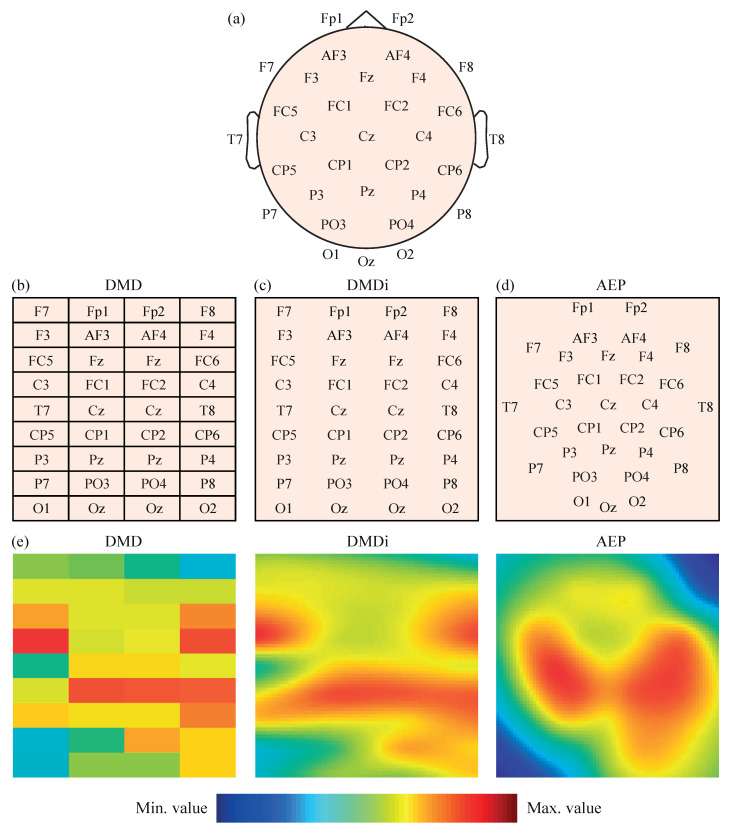
(**a**) Representation of 32 EEG channels on the scalp according to the 10–20 system. (**b**) Distribution of 32 EEG channels in the DMD mapping scheme. (**c**) Distribution of 32 EEG channels in the DMDi mapping scheme. (**d**) Distribution of 32 EEG channels in the AEP mapping scheme. (**e**) Example of $P_{t}$ values from the same segment represented with the three mapping approaches.

The power values $P_{t}$, $P_{\theta }$, $P_{\alpha }$, $P_{\beta }$ and $P_{\gamma }$ obtained for the 32 EEG channels were then initially transformed into 2-D images to feed several CNN models. The original locations of the 32 EEG channels on the scalp presented in Figure 1a were mapped, following three different approaches. The first mapping scheme, called direct matrix distribution (DMD), consisted of the placement of the EEG channels in a 9 × 4 matrix, as shown in Figure 1b. Each element of the matrix directly represented one electrode; thus, the disposition of the channels in the matrix was most similar to their real locations over the scalp, without leaving blank spaces. Furthermore, the channels located between left and right brain hemispheres, i.e., Fz, Cz, Pz and Oz, were duplicated in the central columns of the matrix for a symmetrical representation of all electrodes.

The second mapping approach was called direct matrix distribution interpolated (DMDi). As can be observed in Figure 1c, the DMDi approach presented the same distribution of channels as DMD. The only difference was that DMDi interpolated the power values between channels, making use of a biharmonic spline interpolation scheme [44], and thus coloring the whole 2-D surface. On the contrary, DMD presented a grid in which each cell was filled with a color according to the level of power calculated for the corresponding channel.

The third mapping approach was based on the azimuthal equidistant projection (AEP) [45]. Considering the human head as a sphere, the spherical coordinates of each electrode can be converted into Cartesian coordinates. Therefore, locations in the space were projected over a 2-D surface, maintaining proportional distances between electrodes and directions from the central point of the head, which corresponds to channel Cz. This projection of electrodes in a 2-D map is shown in Figure 1d. A biharmonic spline interpolation was also applied to calculate the values between channels.

It should be noted that, in the three mapping approaches, the spectral power values were represented using a Jet colormap with 256 colors, ranging from dark blue (assigned to the minimum value) to dark red (assigned to the maximum value). Furthermore, given that the input layer of AlexNet requires a 227-by-227 input image [46] (as will be mentioned in the next subsection), the 2-D maps obtained by the three mapping approaches were appropriately rescaled. In the cases of DMDi and AEP, each pixel of the image had a different value due to interpolation. On the other hand, DMD images still maintained a 9 × 4 grid of cells, where each one covered a 25 pixel height (227/9) and 56 pixels width (227/4) with the same color. As an example, Figure 1e shows the same EEG segment represented with the three mapping approaches.

Finally, in order to explore complementary information between frequency sub-bands, 3-D images were also constructed by stacking the resulting 2-D maps from each of the three mapping approaches separately. More precisely, for every EEG excerpt, the 2-D images obtained from the parameters $P_{t}$, $P_{\theta }$, $P_{\alpha }$, $P_{\beta }$ and $P_{\gamma }$ were piled to obtain 3-D cubes with a size of 227 × 227 × 5.

### 2.5. AlexNet-Based CNN Models

AlexNet is the first documented large-scale, pre-trained CNN architecture, which won the ImageNet Large-Scale Visual Recognition Challenge in 2012 [46]. Since then, AlexNet has been used in a variety of applications [25]. The original model was formed by eight layers (five convolutional and three fully connected ones) with weights and ability to learn. On the one hand, convolutional layers assist in the extraction of features, performing a convolution operation between the input data and a kernel with different settled weights. During the training process, a backpropagation algorithm regulates weights according to the target using a rectifier linear unit (ReLU) as an activation function. Note that data resulting from these layers are usually normalized and downsampled using a max-pooling operation to reduce the spatial dimension of the feature map, while retaining the relevant information and making the network less prone to overfitting [46]. On the other hand, the fully connected layers ensure that all the neurons in the previous layer are connected to all neurons in the current one, such that the number of fully connected neurons in the final layer defines the number of output classes.

**Figure 2:**
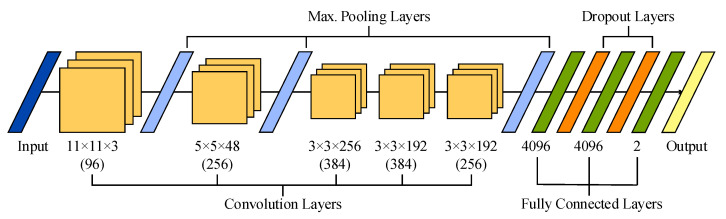
Illustration of the sequential layer-based architecture of AlexNet used in this study.

The original architecture of 2-D AlexNet is presented in Figure 2. This network receives 2-D images with a size of 227 × 227 and three color channels as input data, which are convolved with 96 kernels of size 11 × 11 × 3. Next, the output is normalized and max-pooled before being transmitted to the second convolutional layer, which filters the resulting feature space with 256 kernels of size 5 × 5 × 48. Then, the feature space is also normalized and max-pooled before being filtered with 384 kernels of size 3 × 3 × 256. The resulting feature space is convolved, with two layers presenting similar kernel sizes of 3 × 3 × 192 and 384 and 256 kernels, respectively. No pooling and normalization operations are applied between the third and fifth convolutional layers. Furthermore, a ReLU function is used after every convolutional and fully connected layer. Finally, the resulting feature space is recombined using three fully connected layers with 4096 neurons each. It is worth noting that a dropout regularization operation is performed after the first two layers, randomly dropping out nodes during the training stage with the aim of decreasing overfitting and improving generalization errors [47].

Recently, this 2-D network has been extended to deal with 3-D cubes as input data [48,49]. This 3-D version of AlexNet has been achieved by modifying its original convolution and max-pooling layers. More precisely, the five convolutional layers maintained 96, 256, 384, 384 and 256 kernels, but with sizes of 11 × 11 × 11 × 3, 5 × 5 × 5 × 96, 3 × 3 × 3 × 256, 3 × 3 × 3 × 384, and 3 × 3 × 3 × 384, respectively. Similarly, the size of the three max-pooling layers was extended to 3 × 3 × 3.

### 2.6. Fine-Tuning and Learning Parameters of the AlexNet-Based Models

The original 2-D AlexNet was initially trained with a subset of the ImageNet database, composed by more than one million images, to discern among more than 1000 classes [46]. In the present work, the original layer structure of 2-D AlexNet was adopted, except for the last outcome layer, which was modified to deal with only two classes (i.e., calm and distress), as can be observed in Figure 2. Nonetheless, two different training schemes for this network were conducted. On the one hand, a pre-trained version of AlexNet was fine-tuned by taking advantage of TL. Thus, the original weights of the AlexNet were transferred to this study and fine-tuned during the training stage. On the other hand, a 2-D AlexNet network with initial random weights was trained from scratch. In both cases, training was conducted with 40 epochs, 103 learning iterations per epoch, and a mini batch size of 12 signals. For that purpose, a stochastic gradient descent algorithm with a momentum of 0.9 was used. Furthermore, the initial learning rate was set to a constant value of 0.001 during the 4120 total iterations and no learn rate drop factor was established during the training stage. Additionally, L2 weight decay for convolutional weights was set to 0.001 and both weights and bias were updated at each 103 iterations in the direction of the negative gradient of the loss [50]. To avoid overfitting, the training progress was continuously monitored, validating the network every 50 iterations by predicting the response of the test data and calculating the loss and global accuracy on training and test samples. Thus, it was seen that training loss outcomes were comparable with test loss in every checkpoint, while global training accuracy was not significantly higher than test accuracy.

In a similar way, two 3-D AlexNet models were also trained in this study. Firstly, the weights from the original pre-trained 2-D AlexNet network were stacked five times to deal with the 3-D cubes generated, as described in Section 2.4. Secondly, random weights were initially established in the 3-D network. For both cases, the training stage was conducted as aforementioned for the 2-D AlexNet models, but 250 epochs with 50 iterations per epoch were run.

### 2.7. Experimental Setup and Performance Analysis

Every set of 2-D images obtained from each frequency sub-band, i.e., θ, α, β and γ bands as well as the band covering 4–45 Hz, and from each mapping scheme proposed to distribute the EEG channels into 2-D maps, i.e., DMD, DMDi, and AEP, were used to train the two previously described 2-D AlexNet models. Similarly, the set of 3-D cubes obtained for the three mapping approaches were employed to train the 3-D AlexNet networks with known and random initial weights. To quantify the performance of the resulting CNN model in each case, several validation cycles following a 80/20 hold-out approach were conducted. More precisely, 10 iterations were run for each network, such that, in each one, 658 calm images (out of 822) and 586 distress images (out of 732) were randomly selected for training. Then, the remaining images from each class were used to test the model and values of sensitivity (Se), specificity (Sp) and accuracy (Acc) were obtained. Finally, mean and standard deviation (std) of these performance metrics for the 10 iterations were computed. While Se was defined as the rate of correctly classified distress EEG segments, Sp was defined as the percentage of properly identified calm EEG segments. Finally, Acc was computed as the total proportion of correctly detected EEG segments. These metrics were mathematically computed as

(6) $$Se=\frac{TP}{TP+FN},$$

(7) $$Sp=\frac{TN}{TN+FP},\;\mathrm{and}$$

(8) $$Acc=\frac{TN+TP}{TN+TP+FN+FP},$$

where TP was the number of correctly identified distress EEG segments, TN was the amount of correctly classified calm EEG segments, FP the number of calm segments improperly classified as distress ones, and FN the amount of distress EEG intervals wrongly identified as calm segments.

## 3. Results

### 3.1. AlexNet-Based 2-D CNN Models

Table 1 summarizes the classification outcomes obtained by pre-trained 2-D AlexNet networks for each mapping methodology, described in Section 2.4, and each frequency sub-band analyzed. As can be observed, notable differences in classification were obtained among the different frequency bands. On the one hand, $P_{\theta }$, $P_{\alpha }$ and $P_{\beta }$ achieved comparable global accuracy, ranging from 58.74% to 62.35% when discriminating between calm and distress images. In this respect, DMDi mapping showed a slightly higher performance for $P_{\theta }$ and $P_{\beta }$ bands, almost similar to the score achieved by $P_{\alpha }$ using DMD mapping. However, DMD exhibited lower dispersion among validation cycles than DMDi and AEP mappings. In this respect, values lower than 8% were reported for all the performance indices with DMD representation, whereas higher values of std were reported for DMDi and AEP schemes, especially for the Sp index. On the contrary, std values lower than 3% were reported for Acc, regardless of the mapping scheme used. It is worth noting that average Se and Sp metrics were unbalanced on these frequency sub-bands, showing the same trend for the three mapping approaches. Indeed, the ability to detect correctly distress images was between 10 and 15% higher than that used to detect calm images in α and β sub-bands, and around 30% higher for θ sub-band, when DMD and DMDi mapping schemes were used.

Conversely, the performance shown by the γ sub-band was notably higher than in the other frequencies. Firstly, average accuracies were comparable, ranging from 81.3% for DMDi mapping to 82.6% for AEP mapping. This improvement represents an increase of more than 20% with respect to the other frequency sub-bands. Secondly, std values lower than 3% were observed for Se and Sp and than 1% for Acc. Finally, Se and Sp metrics were well-balanced, reporting differences lower than 3%. The average performance outcomes obtained when the whole frequency band was considered ($P_{t}$) were very similar to those obtained by $P_{\gamma }$. However, the global accuracy increased by 2%, reaching a final value of 84.77% when DMD mapping was used.

Regarding the 2-D AlexNet-based CNN models with random initial weights, Table 2 summarizes the main classification outcomes in terms of mean and std for the 10 validation cycles. In general terms, lower values of Acc than the pre-trained CNN models were provided for all the images obtained from the diverse frequency sub-bands and mapping approaches. More precisely, decreases of about 1 and 2% in values of Acc were noticed for $P_{\theta }$, $P_{\alpha }$ and $P_{\beta }$ when the DMD mapping was used. The decrease in Acc was still more notable for the AEP mapping, reaching values between 2 and 4% lower than with the pre-trained 2-D networks. Moreover, it is worth noting that average values of Se and Sp were significantly unbalanced, especially when the AEP mapping was used. For this mapping approach, high std values among the 10 validation cycles were noticed in terms of Se and Sp for most of the frequency sub-bands.

The same falling trend was also noticed in the classification performance exhibited by $P_{\gamma }$. In this case, Acc values of 76.85%, 77.38% and 77.90% were obtained for DMD, DMDi and AEP mappings, which represented decreases of 6, 4 and 5%, respectively, in comparison with those obtained by the pre-trained 2-D AlexNet-based CNN networks. Nonetheless, in this frequency sub-band, values of Se and Sp were balanced for the three mappings, and the dispersion among validation cycles was notably lower than in $P_{\theta }$, $P_{\alpha }$ and $P_{\beta }$. As in the case of the pre-trained CNN models, the outcomes obtained for the frequency sub-band covering 4–45 Hz were similar to those reported by the γ sub-band. Thus, a poorer performance between 2 and 7% was noticed in this case.

### 3.2. AlexNet-Based 3-D CNN Models

The classification results obtained by 3-D AlexNet-based CNN networks, both with initial weights transferred from the pre-trained 2-D AlexNet (i.e., making use of TL) and with random initial weights (i.e., without using TL), are presented in Table 3 for the three mapping approaches. No great differences were noticed among DMD, DMDi and AEP mapping schemes, but, in both cases, the best results were reported by the first mapping approach. It is also interesting to note that about 3% higher values of Acc were always obtained when 3-D CNN models were initialized with the weights transferred from the original pre-trained 2-D AlexNet. Moreover, lower values of std among the 10 validation cycles, as well as more balanced values of Se and Sp (especially for DMDi and AEP mapping schemes), were also noticed in that case.

## 4. Discussion

The understanding of how emotions are generated and regulated has become the focus of interest in recent years, fostering the appearance of what is known as the science of emotion regulation [51]. In the literature, a wide range of works which face the challenge of emotion recognition from different perspectives can be found. Nonetheless, to the best of our knowledge, this is the first work focused on distress identification from spectral features of the EEG signal with 2-D and 3-D CNN-based models. The results obtained corroborate the hypothesis that evaluating all brain locations simultaneously with a CNN-based classifier represents an enhancement of the outcomes with respect to the analysis of single and isolated EEG channels, traditionally conducted with machine learning algorithms. To this respect, Table 4 shows information from recent studies dealing with stress recognition using EEG spectral features and machine learning classifiers. As can be observed, the classification results obtained by these works presented accuracy values between 57 and 80% [15,16,17,18,52], which are significantly lower than those obtained in the present study by all pre-trained 2-D and 3-D AlexNet-based networks.

It is important to remark that these studies must be compared with caution, because most of them used different methodologies and experimental protocols, where substantial changes in the number of participants, EEG electrodes, and emotion elicitation ways were found. Nonetheless, two studies included in Table 4, i.e., works [15,52], used the same public database as in the present work, and thus a direct and fair comparison could be established. In this case, the use of pre-trained 2-D and 3-D CNN-based classification models has reported an improvement of around 10–15% with respect to the accuracy obtained by common machine learning classifiers in [15,52]. This outcome is in line with previous studies where deep learning methods have outperformed the results reported by traditional machine learning classifiers, such as SVM and decision trees, for the recognition of different valence and arousal levels [53].

For the three mapping approaches, the best classification outcomes using 2-D CNN-based networks were reported by the relative power obtained from the frequency band covering 4–45 Hz, i.e., $P_{t}$. The relevance of this power measure with respect to the sub-bands θ, α, β and γ has also been previously described in another study of emotion recognition from EEG signals with CNNs [54]. This could be explained by the fact that these frequency sub-bands are involved in emotional processes, although with a different degree of significance depending on the emotional state. Indeed, classification results obtained by $P_{\gamma }$ were notably higher than those obtained by the remaining sub-bands. Thus, an improvement of more than 20% of accuracy with respect to $P_{\theta }$, $P_{\alpha }$ and $P_{\beta }$ was noticed for the three mapping schemes, when discerning between calm and distress. The brain activity in the sub-band γ has already been strongly related to emotional processes, with a special intensity for negative stimuli [55]. Indeed, the induction of negative emotions has reported a higher level of activity in this sub-band with respect to positive and neutral stimuli in healthy subjects [56,57]. Moreover, in previous works $P_{\gamma }$ has also reported a notable increment in frontal and parietal regions under stressful conditions during task-switching activities [58]. The waves γ reflected on the EEG have also allowed for the differentiation of worry from relaxation and baseline, with an increase in activity in the sub-band γ for the case of patients with anxiety disorder [59].

In the literature, other research has also applied 2-D CNN-based approaches for the classification of different emotions by means of EEG spectral features using similar mapping approaches. In this respect, Li et al. [19] used a mapping scheme similar to DMDi for the representation of spectral power values from four different emotional states. The maximum classification accuracy ranged between 50% and 75%, depending of the window time size selected to estimate spectral features from the EEG channels, and using a combination of CNN and long–short-term memory recurrent neural networks [19]. On the other hand, Li et al. [60] represented spectral features in AEP maps for the identification of depression using different CNN-based classifiers. The accuracy outcomes obtained for the spectral power of the whole frequency band ranged between 76 and 80%, depending on the hyper-parameters used in each CNN model [60]. Although comparisons should be carefully established, the present study outperformed the results from these works, achieving accuracy values of about 85%. This improvement could be a consequence of the different focuses of these works, since the emotional states under study are different in each case.

Another strength of the present work regarding the aforementioned studies is the use of pre-trained AlexNet-based networks, instead of CNN-based models constructed and trained from scratch [19,60]. According to the literature, the use of TL methodologies and fine-tuning parameters to adapt pre-trained networks to different problems could have some advantages over networks constructed from scratch [34]. On the one hand, the number of samples needed to train a CNN from scratch is large due to the high number of parameters that need to be settled. Instead, TL provides an effective way of training complex network architectures using scarce data without overfitting [61]. Furthermore, hyper-parameters have to be configured randomly and then readjusted when a CNN is constructed from scratch, thus requiring extensive computational and memory resources [62]. On the contrary, hyper-parameters only have to be refined when using pre-trained networks. Therefore, most weights are maintained and only a few are filtered to adapt the network to the current classification problem, thus achieving better generalization and outperforming other models constructed from scratch. Accordingly, the results obtained in the present work were better when TL was used to maintain initial weights in pre-trained 2-D and 3-D AlexNet-based networks than when random initial weights were established in similar classifiers, regardless of the mapping approach. Thus, in addition to obtaining values of Acc between 3 and 6%, better, more balanced values of Se and Sp and lower dispersion among validation cycles were noticed in the first case.

To assess the criticality of representing neural information in 2-D images used as input data for CNN-based classifiers, three different mapping approaches have been evaluated. All DMD, DMDi, and AEP maps preserved the topology information in EEG channels, since they were distributed to resemble the actual location of the electrodes over the scalp. The difference is that DMD and DMDi were a direct reorganization of all EEG channels in a 9 × 4 matrix, without considering their relative locations, and the AEP model was a projection of the electrodes into a 2-D image. In this last case, the proportions of distance and direction of the EEG channels with respect to a reference point were regarded. In terms of classification performance, the results obtained by the three mapping approaches in each frequency sub-band were similar, with slight differences in values of Acc around 1–3%. Consequently, the spatial information provided by preservation of proportional locations of the EEG channels within AEP images seems not to imply a notable improvement regarding the matrix distribution of the electrodes in DMD and DMDi maps. Furthermore, interpolation between electrodes in the matrix distribution did not entail substantial changes between classification results reported by DMD and DMDi maps. As a result, selection of one of these three mapping approaches would not be crucial to discern between emotional states of calm and distress from EEG recordings. A similar finding has also been obtained in a previous work that compares different mapping approaches to detect high and low levels of arousal and valence with a CNN-based classifier [63]. More precisely, in that work, four matrix distributions with random positions of the EEG channels and two DMD-based schemes were analyzed [63]. No relevant differences were obtained among the six mapping options, thus suggesting that the distribution of EEG channels into 2-D maps does not play a key role in identifying different emotional processes [63]. On the contrary, the presence of information from all EEG channels, instead of only single locations, could be more essential than the distribution of the electrodes in the images.

Similarly, no great improvement in distress detection was noticed when 2-D images were stacked to construct 3-D cubes with simultaneous information from all analyzed frequency sub-bands (i.e., θ, α, β, γ, and the whole frequency band covering 4–45 Hz). Indeed, for the three mapping approaches, 3-D AlexNet-based CNN networks only reported values of Acc 1–2% greater than 2-D classifiers. This result was observed both when the networks maintained initial weights from previous pre-training and when they used random initial weights. The fact that the sub-band γ seems to contain the most relevant information to discern between emotional states of calm and distress could explain that outcome. Indeed, 2-D AlexNet-based classifiers have provided no relevant classification differences between using 2-D maps from the sub-band γ or from the frequency band covering 4–45Hz, thus suggesting that no complementary information exists among the analyzed spectral regions.

To bring any of our CNN-based systems to the real world, we will embed it into a programmable logic device. Although it is well-known that CNN-based algorithms are computationally intense and require vast computational resources and dynamic power for computation of convolutional operations, in recent years, some programmable devices have been specifically developed to run these kinds of algorithms in real-time [64]. In this respect, some researchers have already successfully tested a variety of hardware implementation methods for different CNN-based structures, mostly based on field-programmable gate arrays (FPGA) architectures [65,66]. In fact, at present there are available commercial FPGA-based systems designed for vision artificial intelligence, which can be configured for enhancing the acceleration of vision applications. Interestingly, they contain implemented CNN-based algorithms which can be adapted and tuned for diverse applications [67]. Hence, bearing in mind that technical requirements of our system would be considerably limited regarding those needed by common vision artificial intelligence applications, its implementation in such embedded devices will be feasible. Indeed, our system only analyzes a 227 × 227 × 3 image each five seconds, and its computational load can, therefore, be considered notably low in comparison with other applications requiring real-time image processing with CNN-based methodologies.

Finally, some limitations should be considered. Firstly, every CNN-based algorithm does not allow for understanding the rationale behind its classification results. Thus, it is not possible to give a clinical interpretation of the outcomes, since functional dependencies between input and output information are completely hidden [68]. On the other hand, the database from which the EEG signals were extracted was not specifically created for recognition of calm and distress, since it contains samples corresponding to emotions in the whole valence/arousal space. Moreover, although the DEAP database presents interesting advantages, such as it being freely available and able to obtain comparable results with other previous studies, it contains a limited number of subjects. Additionally, the number of calm and distress samples for each subject is not completely balanced, and some subjects do not present samples from both emotional states. These two aspects aimed for all samples to be considered together for training and testing the CNN-based models, regardless of the subject they came from, such as in some previous works [29]. Nonetheless, because this approach could lead to some overfitting, additional experiments considering separate subjects for training and testing every classifier were also conducted. In this respect, the pre-trained 2-D and 3-D AlexNet-based CNN networks were trained and tested through 10 validation cycles, where, in each one, the participants randomly selected for training had approximately the 80% of the samples, and the remaining ones were used for testing. In this case, for the three mapping approaches, only values of Acc about 3–4% and 1–2% lower than those presented in Section 3 were noticed for pre-trained 2-D and 3-D classifiers, respectively, thus suggesting that the proposed algorithms for distress recognition were not significantly overtrained.

## 5. Conclusions

The present work has introduced, for the first time, the use of 2-D and 3-D CNN-based classifiers to discern emotional states of calm and distress by taking advantage of the brain’s behavior as a synchronized network. Thus, receiving 2-D and 3-D spectral maps constructed from common 32 channel EEG recordings as input data, the algorithms have provided significantly better classification of both emotions than previous methodologies based on combining isolated information from different brain regions with machine learning algorithms. Moreover, the use of pre-trained CNN-based classification models has also improved the diagnostic accuracy reported by other similar deep learning algorithms trained from scratch, thus highlighting the usefulness of transfer learning in distress recognition. Finally, no significant differences between the results provided by the three approaches considered to reconstruct 2-D spectral maps were noticed, then suggesting an independency of the distribution of the EEG electrodes in the maps.

## Figures and Tables

**Table 1:** Classification results reported by pre-trained AlexNet networks for 2-D images obtained from $P_{\theta }$, $P_{\alpha }$, $P_{\beta }$, $P_{\gamma }$ and $P_{t}$ using DMD, DMDi and AEP mapping schemes.

		DMD			DMDi			AEP		
		**Se (%)**	**Sp (%)**	**Acc (%)**	**Se (%)**	**Sp (%)**	**Acc (%)**	**Se (%)**	**Sp (%)**	**Acc (%)**
**$P_{\theta }$**	Mean	77.26	43.36	61.29	74.76	48.42	62.35	66.95	53.49	60.61
	Std	2.42	3.17	2.43	3.35	11.48	1.75	13.68	16.83	2.07
**$P_{\alpha }$**	Mean	65.30	56.16	61.00	68.23	52.67	60.90	65.12	51.58	58.74
	Std	3.58	7.15	0.42	12.79	19.20	1.54	4.49	4.32	0.09
**$P_{\beta }$**	Mean	68.29	50.48	59.90	63.23	58.97	61.23	61.95	59.79	60.94
	Std	6.45	7.52	1.05	3.93	6.74	0.48	10.16	12.76	1.02
**$P_{\gamma }$**	Mean	81.8	83.2	82.4	79.9	82.9	81.3	82.5	82.7	82.6
	Std	1.98	2.77	0.69	1.49	1.37	0.45	2.18	2.23	0.53
**$P_{t}$**	Mean	85.24	84.25	84.77	80.67	82.60	81.58	83.66	84.52	84.06
	Std	0.62	3.02	0.44	1.52	2.86	0.66	0.39	0.95	0.35

**Table 2:** Classification results reported by AlexNet networks with random initial weights for 2-D images obtained from $P_{\theta }$, $P_{\alpha }$, $P_{\beta }$, $P_{\gamma }$ and $P_{t}$ using DMD, DMDi and AEP mapping schemes.

		DMD			DMDi			AEP		
		**Se (%)**	**Sp (%)**	**Acc (%)**	**Se (%)**	**Sp (%)**	**Acc (%)**	**Se (%)**	**Sp (%)**	**Acc (%)**
**$P_{\theta }$**	Mean	69.13	47.77	59.07	80.95	32.11	57.94	81.48	29.20	56.85
	Std	10.71	11.18	1.25	11.37	29.33	1.44	20.22	28.48	1.39
**$P_{\alpha }$**	Mean	74.70	44.86	60.65	74.70	44.86	60.60	87.04	21.32	56.09
	Std	13.20	39.65	2.27	13.20	39.65	2.27	14.48	39.41	1.65
**$P_{\beta }$**	Mean	55.49	64.73	59.84	71.49	39.73	56.53	61.20	53.00	57.34
	Std	8.31	9.50	0.72	21.32	38.92	0.66	5.43	10.86	0.48
**$P_{\gamma }$**	Mean	76.83	76.88	76.85	80.56	73.80	77.38	75.84	80.22	77.90
	Std	3.64	6.18	0.22	1.14	1.31	1.54	0.20	1.58	0.29
**$P_{t}$**	Mean	76.75	80.91	78.71	80.03	79.79	79.92	76.83	76.88	76.85
	Std	2.29	1.43	0.18	1.11	4.75	2.29	3.64	6.18	0.23

**Table 3:** Classification results reported by AlexNet-based CNN networks with initial weights transferred from the pre-trained 2-D AlexNet and with random initial weights for 3-D cubes obtained with DMD, DMDi and AEP mapping schemes.

Initials Weights		DMD			DMDi			AEP		
		**Se (%)**	**Sp (%)**	**Acc (%)**	**Se (%)**	**Sp (%)**	**Acc (%)**	**Se (%)**	**Sp (%)**	**Acc (%)**
Known	Mean	87.11	84.77	86.12	85.25	84.32	84.87	83.48	85.25	84.18
(with TL)	Std	0.71	1.18	1.25	1.37	1.77	1.44	0.88	1.48	1.39
Random	Mean	82.42	83.27	82.87	77.32	84.71	81.23	75.32	85.15	80.52
(without TL)	Std	5.39	6.90	4.05	3.42	2.86	3.6	3.42	4.31	2.14

**Table 4:** Comparison of the present study with other works detecting stress from power spectral features of EEG signals and machine learning classification algorithms.

Work	Experiment	Classifier	Results
Jebelli et al. [16]	7 subjects 14 EEG channels Construction work	SVM	Max Acc: 80.32%
Shon et al. [15]	32 subjects 32 EEG channels Videoclips	k-NN	Max Acc: 71.76%
Ahn et al. [17]	7 subjects 2 EEG channels Eyes open and close	SVM	Max Acc: 77.90%
Arsalan et al. [18]	28 subjects 4 EEG channels Resting pre and post-activity	SVM, NB ${}^{1}$ and MLP ${}^{2}$	57–71% with all frequency bands
Hasan & Kim [52]	32 subjects 32 EEG channels Videoclips	k-NN	Max Acc: 73.38%
This work	32 subjects 32 EEG channels Videoclips	2-D AlexNet 3-D AlexNet	Max Acc: 84.77% Max Acc: 86.12%

^1^ NB: Naive Bayes; ^1^ MLP: Multilayer perceptron.
